# Validation of a Portuguese version of the health-related quality of life measure for active chronic otitis media (COMQ-12)^☆^

**DOI:** 10.1016/j.bjorl.2017.08.007

**Published:** 2017-09-01

**Authors:** Anna Carolina Oliveira Fonseca, Pedro Ramos, Fernando A. Balsalobre, Edson L. Freitas, John S. Phillips, Matthew W. Yung, Ricardo F. Bento

**Affiliations:** a Universidade de São Paulo (USP), Faculdade de Medicina, Departamento de Otorrinolaringologia, São Paulo, SP, Brazil; b Universidade de São Paulo (USP), Faculdade de Medicina, Hospital das Clínicas, São Paulo, SP, Brazil; c Norfolk & Norwich University Hospital, Department of Otolaryngology-Head and Neck Surgery, Norwich, United Kingdom; d Ipswich Hospital, Department of Otolaryngology-Head and Neck Surgery, Ipswich, United Kingdom

**Keywords:** Chronic suppurative otitis media, Otology, Patient-reported outcome, Questionnaire, Quality of life, Otite média supurativa crônica, Otologia, Resultado relatado pelo paciente, Questionário, Qualidade de vida

## Abstract

**Introduction:**

Measuring the impact on quality of life, especially after the beginning of the treatment, is becoming increasingly important in healthcare.

**Objective:**

The aim of this study was to translate the Chronic Otitis Media Questionnaire-12 (COMQ-12) into Portuguese language and validate this version in a group of patients with chronic otitis media.

**Methods:**

The Portuguese version of COMQ-12 was obtained by translation and back translation. Portuguese speaking patients with a history of active chronic otitis media were asked to complete the COMQ-12 Portuguese version. Cronbach's *α* coefficient was calculated for an estimation of the internal consistency of the questionnaire.

**Results:**

A total of 100 patients were included in the study; 49 women and 51 men, with a mean age of 39 years (range 12–77 years, median 40 years). The average COMQ-12 score was 29, out of a maximum score of 60. Cronbach's *α* result for the Portuguese version of the COMQ-12 was 0.85, indicating a high internal consistency. The participants presented with different forms of chronic otitis media, and almost all domains of the COMQ-12 questionnaire were able to differentiate between patients with healed chronic otitis media and patients with cholesteatoma or wet tympanic membrane perforation. Showing that patients with healed chronic otitis media have a better quality of life, measured by the COMQ-12, is a first step to guarantee the questionnaire's validity. The next step will consist on routinely using the questionnaire in patients undergoing surgery for chronic otitis media in order to evaluate their quality of life after treatment.

**Conclusion:**

The COMQ-12 Portuguese version showed high reliability, and may be used as an assessment of quality of life in patients with chronic otitis media.

## Resumo

**Introdução:**

Medir o impacto na qualidade de vida, especialmente após o início do tratamento dos pacientes, está se tornando cada vez mais importante nos cuidados da saúde.

**Objetivo:**

O objetivo deste estudo foi traduzir o Questionário de Otite Média Crônica-12 (COMQ-12) para a língua portuguesa e validar essa versão em um grupo de pacientes com Otite Média Crônica.

**Método:**

A versão em Língua Portuguesa do COMQ-12 foi obtida através de tradução e posterior retrotradução. Pacientes nativos da língua portuguesa com histórico de OMC ativa foram convidados a completar o COMQ-12 em Português. O coeficiente α de Cronbach foi calculado para estimar a consistência interna do questionário.

**Resultados:**

Um total de 100 pacientes foram incluídos no estudo; 49 eram mulheres e 51 eram homens, com média de idade de 39 anos (variação: 12 a 77 anos, mediana de 40 anos). O escore médio do COMQ-12 foi 29, de um escore máximo de 60. O resultado do coeficiente α de Cronbach para a versão em português do COMQ-12 foi de 0,85, indicando que sua consistência interna era alta. Os participantes apresentavam diferentes formas de otite média crônica e quase todos os domínios do questionário COMQ-12 foram capazes de diferenciar entre pacientes com otite média crônica curada e pacientes com colesteatoma ou perfuração úmida de membrana timpânica. Demonstrar que pacientes com otite média crônica curada apresentam uma melhor qualidade de vida, medida pelo COMQ-12 é o primeiro passo para garantir a validade do questionário. O próximo passo será utilizá-lo rotineiramente em pacientes submetidos à cirurgia para otite média crônica e avaliar a qualidade de vida após o tratamento.

**Conclusão:**

A versão em português do questionário COMQ-12 mostrou alta confiabilidade e pode ser utilizada como questionário de medida de qualidade de vida em pacientes com otite média crônica.

## Introduction

Chronic Otitis Media (COM) is characterized by inflammation of the middle ear that may result in long-term or permanent changes in the tympanic membrane.^1^ Different forms of COM include perforation, atelectasis, retraction, tympanosclerosis, and cholesteatoma. The global estimated burden of disease from COM varies widely worldwide, with prevalence rates ranging from <1% in high-income countries to >4% in low-income countries.^2^ The disease may be associated with significant morbidity, mostly in resource-limited settings, and a high impact on patients’ quality of life with a weight of over 2 million DALYs (Disability-Adjusted Life Years).^3^ Measuring this impact on quality of life, specially after patients start treatment, is becoming increasingly important in healthcare. Patient-Reported Outcome Measures (PROMs) are standardized questionnaires that aim to capture this effect on Health-Related Quality of Life (HRQoL), by including measures of symptom status, physical function, mental health, social function, and wellbeing.^4^ The Chronic Otitis Media Questionnaire-12 (COMQ-12) is a disease-specific questionnaire for the assessment of the quality of life in patients with COM that may be used as a PROM.^5^, ^6^ To date, it has been translated and validated into two languages,^7^, ^8^ which provides a common cross-cultural measure of HRQoL for comparative and multicenter studies.

The aim of the present study was to translate the Chronic Otitis Media Questionnaire-12 (COMQ-12) into Portuguese language and validate this version in a group of patients with complaints of COM.

## Methods

### The questionnaire

The COMQ-12 is a health-related quality of life questionnaire developed by Phillips et al.,^5^ composed of 12 self-rating questions grouped in four categories. Each question needs to be scored on a six-point ordinal scale from 0 (i.e., no impact) to 5 (i.e., most severe impact). Questions 1–7 are related with the severity of symptoms. Questions 8 and 9 ask about the impact on work and lifestyle; questions 10 and 11 look at the impact on the health service, and question 12 is about the general impact on the patient.

### The translation process

The Portuguese version of COMQ-12 was obtained with permission of the original authors and followed the standardized process for PROM translation.^9^

The English version of COMQ-12 was translated independently by 2 native Portuguese-speaking otolaryngologists with proficient knowledge of English. A bicultural expert compared the two translated questionnaires and produced a final version. The Portuguese version was then translated back into English by one native English-speaking. There were no conceptual differences in the back-translation compared to the original version. The final Portuguese version of the COMQ-12 was then approved in format and content (available in the supplemental information section).

### Ethical considerations

Ethics committee approval (n° 1.887.778) was received for this study according to the Declaration of Helsinki and informed consent was obtained from all participants.

### Subjects

Portuguese speaking patients with a history of active COM were asked to complete the Portuguese version of the COMQ-12. Inclusion criteria required that patients were older than 12 y.o. and had a history of active COM of more than 6 months. The questionnaire was completed by 100 patients with different types of COM. Additionally, we also collected some clinical and demographical information for each patient.

### Statistical methods

To estimate the internal consistency of the Portuguese version of the COMQ-12, Cronbach's *α* coefficient was calculated. This index is used to measure the questionnaire reliability. Internal consistency is considered satisfactory if Cronbach's *α* reaches 0.7, but ≥0.8 is recommended.

We also analyzed the questionnaire's construct validity using the method of known-groups validity. Known-groups validity consists of showing that the instrument may differentiate between groups of patients with different stages or manifestations of the disease, in this case between patients with healed COM (i.e., dry ear) (intact TM, neotympanum, tympanosclerosis) and patients with cholesteatoma or patients with active mucosal COM (wet perforation).

The data were analyzed using STATA^®^ version v13.0 (StataCorp LP, USA).

## Results

A total of 100 patients were included in the study; 49 were women and 51 were men, with a mean age of 39 y.o. (range 12–77 years, median 40 years). Epidemiological characteristics of the study population are shown in Table 1. 72 patients lived in Sao Paulo (Metropolis), 27 lived outside of the metropolis and only 1 lived outside of the state of Sao Paulo. The time required to complete this questionnaire is approximately 10 min.

**Table 1:** Demographic characteristics of the study population (*n* = 100).

Characteristics	Value
*Age (years)*	
Mean	39
SD	17
Median	40
Range	12–77

*Gender, n (%)*	
Female	49%
Male	51%

*Educational level*	
None	3%
Incomplete primary education	26%
Primary education (complete)	36%
Secondary education	33%
University education	2%

SD, standard deviation.

The participants presented with different forms of COM that are shown in Table 2.

**Table 2:** Description of different manifestations of COM in the study population (*n* = 100).

Description	Total (*n*)
Inactive mucosal COM (dry perforation)	36
Active mucosal COM (wet perforation)	26
Inactive squamous epithelial COM (retraction, atelectasis, epidermosis)	16
Active squamous epithelial COM (cholesteatoma)	20
Healed COM (intact TM, neotympanum, tympanosclerosis)	26

The average COMQ-12 score was 29 (SD = 12.8) range from 6 to 56, out of a maximum score of 60. The descriptive statistics for each question is described in Table 3.

**Table 3:** Score averages and distributions for individual COMQ-12 question responses.

Question	Q1	Q2	Q3	Q4	Q5	Q6	Q7	Q8	Q9	Q10	Q11	Q12
Mean	2.92	2.89	2.66	2.70	2.54	1.97	2.34	1.83	2.47	1.56	1.92	3.31
Median	3	3	3	3	3	1.5	2	1	2.5	2	2	3
SD	1.61	1.70	1.51	1.51	1.65	1.85	1.75	2.02	2.29	1.23	1.92	1.63
Variance	2.57	2.89	2.29	2.29	2.72	3.42	3.05	4.08	5.24	1.52	3.67	2.66

Cronbach's *α* result for the Portuguese version of the COMQ-12 was 0.85, indicating that the internal consistency of the questionnaire was high.

The content validity of the questionnaire was ensured by how the original version was developed, by literature review, and by discussion with experienced otolaryngologists. This thorough process aimed to guarantee that the practical purpose of this instrument was maintained.

**Figure 1:**
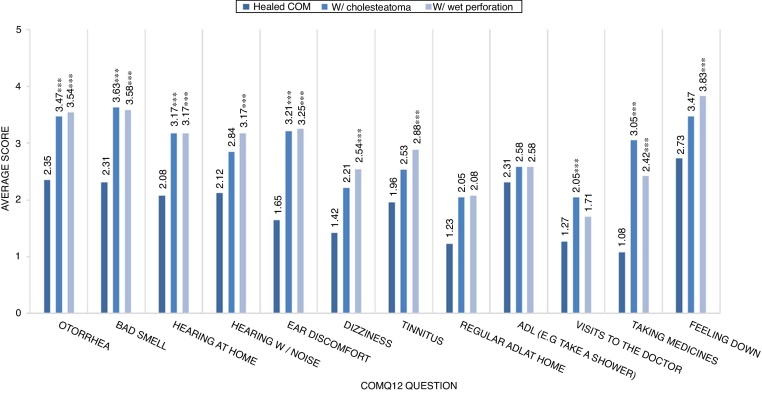
Known-groups validity comparing the questionnaire scores with clinician-rated manifestations of COM. *** indicates that there was a statistical significant difference between that category average score and the “Healed COM” category average score, for that COMQ-12 question.

Known-groups validity was used to compare the questionnaire scores with clinician-rated manifestations of COM (Fig. 1). Almost all domains of the COMQ-12 questionnaire were able to differentiate between patients with healed COM and patients with cholesteatoma or wet perforation. Both conditions were associated with a worsening impact on audition, ear bad smell, ear discomfort, otorrhea, higher frequency of medical visits and need of medication.

## Discussion

The present study translated into Portuguese and validated the COMQ-12 in patients with a history of COM. No major difficulties were found in the translation or the cultural adaptation of the questionnaire. However, the questions were not very easily understood by patients and, therefore, they were sometimes given assistance by the clinical staff in answering to the questionnaire. The majority of the patients that completed the questionnaire (65%) did not have secondary education and only 2% attended University. Therefore, the main concern when applying the questionnaire to this population is to make sure that they understand what is being asked. For this reason, there is already an ongoing study at our center that compares different ways of applying the COMQ-12 to our patients in routine practice. This will be a methodological challenge we will explore soon, and that may give further insights of how COMQ-12 may be used as a PROM tool.^6^

The internal validity of our study was very good, with Cronbach's *α* score of 0.85. This result is in line with the original English version^5^ and a recent Dutch validation study.^7^

As expected, patients that did not have dry ear, namely those with cholesteatoma or with a wet perforation, had significantly higher COMQ-12 scores, compared to patients with healed COM. Furthermore, among healed COM patients the median COMQ-12 score was 2 and the modal score was 0 for 50% of the patients, which is similar to the findings of Kosyakov et al.^8^ and reassures us of our results. Showing that patients with healed COM have a better quality of life measured by the COMQ-12 is a first step to guarantee its validity. The next step will be to use it routinely in patients undergoing surgery for COM and follow-up their quality of life after treatment.

The most worrisome ear-specific problems were dizziness and tinnitus; yet, being limited in daily-life had even lower scores than the reported ear-specific problems, which highlights the true extent of disability in these patients.

Finally, we applied the questionnaire in patients older than 12 y.o. Although the original study used a sample of adult individuals, the COMQ-12 is being gradually applied in younger patients.^7^, ^8^ To the extent that we did not find any differences between patients younger and older than 18 y.o. (data not shown, available upon request), it is one additional evidence toward using the questionnaire in younger patients. Furthermore, the questionnaire was completed in the presence and with the help of younger patients’ legal guardians.

This study has some limitations. Firstly, we recruited a convenience sample of patients. Nonetheless, we believe that this sample adequately represents the population of interest for applying the COMQ-12. Secondly, we did not report test-retest reliability or the responsiveness to change throughout the patients’ treatment.

## Conclusion

The Portuguese version of the COMQ-12 showed high reliability, allowing its clinical use as a HRQoL questionnaire for the assessment of COM.

## Conflicts of interest

The authors declare no conflicts of interest.

## Appendix A Supplementary data

Portuguese version of the Chronic Otitis Media Questionnaire-12 (COMQ-12).
