# Supplementary material for: Validation of a Portuguese version of the health-related quality of life measure for active chronic otitis media (COMQ-12)
Source: Braz J Otorhinolaryngol. 2017 Sep 1;84(6):708–12. doi: 10.1016/j.bjorl.2017.08.007 (PMC9442823; doi:10.1016/j.bjorl.2017.08.007)
Supplement: Supplementary file 1 [file mmc1.pdf]

Questionário de otite média crônica (COMQ-12):

Estas questões são para identificar o quanto (ou “quão mal”) os seus problemas no ouvido te afetam. Nenhuma máquina pode fazer isto: apenas você pode nos contar. Nós esperamos que os resultados vindos deste questionário possam nos ajudar a entender qual sintoma no seu ouvido é mais importante para você. Saber isto irá nos ajudar a melhorar a forma como pacientes com problemas de ouvido são tratados.

Por favor, responda às perguntas abaixo considerando cuidadosamente cada pergunta e então escolhendo o número apropriado; cada número refere-se a uma descrição particular.

Não existem respostas certas ou erradas, mas procure pensar com cuidado sobre cada pergunta antes de escolher o número apropriado. Por favor, considere cada problema da forma como ele se comportou nos últimos 6 meses.

Se você tiver qualquer dificuldade em responder às perguntas, por favor, peça ajuda para algum dos médicos do grupo.

Obrigado.

Para as perguntas seguintes, por favor, indique quão gravemente os vários elementos descritos te afetam, utilizando a escala e marcando o número apropriado:

Severidade do sintoma:

1. **Saída de secreção pelo ouvido** **(ASSINALAR APENAS 1 ALTERNATIVA)**

(0) não me incomoda em nada

(1) é um inconveniente pequeno

(2) é um inconveniente moderado

(3) é um grande inconveniente, mas eu consigo lidar com ele

(4) é um grande inconveniente e eu estou achando difícil de lidar com ele

(5) é a pior coisa que já afetou a minha vida

2. Ter o ouvido com cheiro ruim **(ASSINALAR APENAS 1 ALTERNATIVA)**

(0) não me incomoda em nada

(1) é um inconveniente pequeno

(2) é um inconveniente moderado

(3) é um grande inconveniente, mas eu consigo lidar com ele

(4) é um grande inconveniente e eu estou achando difícil de lidar com ele

(5) é a pior coisa que já afetou a minha vida

3. Problemas de audição em casa, ex. pedir para que o volume do rádio ou TV seja aumentado **(ASSINALAR APENAS 1 ALTERNATIVA)**

(0) não me incomoda em nada

(1) é um inconveniente pequeno

(2) é um inconveniente moderado

(3) é um grande inconveniente, mas eu consigo lidar com ele

(4) é um grande inconveniente e eu estou achando difícil de lidar com ele

(5) é a pior coisa que já afetou a minha vida

4. Problemas de audição ao conversar com grupos de pessoas ou quando o ambiente é barulhento **(ASSINALAR APENAS 1 ALTERNATIVA)**

(0) não me incomoda em nada

(1) é um inconveniente pequeno

(2) é um inconveniente moderado

(3) é um grande inconveniente, mas eu consigo lidar com ele

(4) é um grande inconveniente e eu estou achando difícil de lidar com ele

(5) é a pior coisa que já afetou a minha vida

5. Desconforto dentro e/ou em volta do ouvido (ASSINALAR APENAS 1 ALTERNATIVA)

(0) não me incomoda em nada

(1) é um inconveniente pequeno

(2) é um inconveniente moderado

(3) é um grande inconveniente, mas eu consigo lidar com ele

(4) é um grande inconveniente e eu estou achando difícil de lidar com ele

(5) é a pior coisa que já afetou a minha vida

6. Tontura ou sentir-se desequilibrado (ASSINALAR APENAS 1 ALTERNATIVA)

(0) não me incomoda em nada

(1) é um inconveniente pequeno

(2) é um inconveniente moderado

(3) é um grande inconveniente, mas eu consigo lidar com ele

(4) é um grande inconveniente e eu estou achando difícil de lidar com ele

(5) é a pior coisa que já afetou a minha vida

7. Zumbido ou barulhos no ouvido (ASSINALAR APENAS 1 ALTERNATIVA)

(0) não me incomoda em nada

(1) é um inconveniente pequeno

(2) é um inconveniente moderado

(3) é um grande inconveniente, mas eu consigo lidar com ele

(4) é um grande inconveniente e eu estou achando difícil de lidar com ele

(5) é a pior coisa que já afetou a minha vida

Para as perguntas seguintes, por favor, indique **qual é a frequência** com que os vários elementos descritos te afetam, utilizando a escala e escolhendo o número apropriado.

Estilo de vida e impacto no trabalho:

8. **Com que frequência você NÃO foi capaz de realizar suas atividades diárias normais em casa ou no trabalho (ASSINALAR APENAS 1 ALTERNATIVA)**

(0) menos frequente do que a cada 6 meses

(1) pelo menos 1 vez a cada 6 meses

(2) pelo menos 1 vez a cada 3 meses

(3) pelo menos 1 vez por mês

(4) pelo menos 1 vez por semana

(5) a maioria dos dias da semana

9. **Com que frequência você NÃO foi capaz de lavar-se, tomar banho de chuveiro ou banhar-se como você gostaria? (ex. com que frequência você teve medo de que estas atividades causassem uma infecção no ouvido?) (ASSINALAR APENAS 1 ALTERNATIVA)**

(0) menos frequente do que a cada 6 meses

(1) pelo menos 1 vez a cada 6 meses

(2) pelo menos 1 vez a cada 3 meses

(3) pelo menos 1 vez por mês

(4) pelo menos 1 vez por semana

(5) a maioria dos dias da semana

Impacto no serviço de saúde:

10. **Qual a frequência com que você vai às consultas com o Otorrinolaringologista devido aos seus problemas de ouvido?**
    (ASSINALAR APENAS 1 ALTERNATIVA)

(0) menos frequente do que a cada 6 meses

(1) pelo menos 1 vez a cada 6 meses

(2) pelo menos 1 vez a cada 3 meses

(3) pelo menos 1 vez por mês

(4) pelo menos 1 vez por semana

(5) a maioria dos dias da semana

11. **Qual a frequência com que você precisa tomar medicamentos (incluindo gotas no ouvido) para o seu problema de ouvido?**
    (ASSINALAR APENAS 1 ALTERNATIVA)

(0) menos frequente do que a cada 6 meses

(1) pelo menos 1 vez a cada 6 meses

(2) pelo menos 1 vez a cada 3 meses

(3) pelo menos 1 vez por mês

(4) pelo menos 1 vez por semana

(5) a maioria dos dias da semana

Para a próxima pergunta, por favor **indique o quão mal as coisas são**, em uma escala de ‘0’ a ‘5’.

‘0’ significa **nada**
‘5’ significa o **pior que você pode imaginar**

Geral:

12. **Em que grau os seus problemas de ouvido deixam você “para baixo”? (=desanimado)** **(ASSINALAR APENAS 1 ALTERNATIVA)**

(0) (1) (2) (3) (4) (5)

Por favor, verifique se você respondeu a todas as perguntas e peça ajuda se você tiver alguma dificuldade. Muito obrigado por participar.
